# Volatile Compounds and Fatty Acids of Mutton Carrot Filling During Dynamic Steaming Investigated Based on GC-MS and GC-IMS Analyses

**DOI:** 10.3390/foods14091535

**Published:** 2025-04-27

**Authors:** Kaiyan You, Qianyu Li, Ya Wang, Xuehui Cao

**Affiliations:** College of Food Science and Technology, Bohai University, Jinzhou 121013, China; 2021015071@qymail.bhu.edu.cn (K.Y.); 2022015118@qymail.bhu.edu.cn (Q.L.); 2022015048@qymail.bhu.edu.cn (Y.W.)

**Keywords:** SPME-GC-MS, GC-IMS, mutton, volatile compounds, fatty acid

## Abstract

To investigate the impact of varying steaming durations on the flavor characteristics of mutton and carrot stuffing, dynamic changes in volatile organic compounds (VOCs) and fatty acids were analyzed using solid-phase micro-extraction gas chromatography–mass spectrometry (SPME-GC-MS) and gas chromatography–ion mobility spectrometry (GC-IMS). The results revealed a total of 116 VOCs identified throughout the steaming process, with 73 detected by GC-MS and 44 by GC-IMS. Notably, VOC concentrations were significantly higher at 18–24 min compared to 8–16 min. Additionally, a GC-IMS fingerprint was developed to assess the distribution of VOCs during steaming. Orthogonal partial least squares discriminant analysis (OPLS-DA) indicated that 11 compounds, such as ethyl caprylate (B3), linalyl acetate (B6), and 1-nonanal (C1), significantly influenced the flavor characteristics of the mutton and carrot filling. Further analysis demonstrated that stearic acid content reached its lowest point at 20–22 min of steaming, while n-6 and n-3 series polyunsaturated fatty acids (PUFAs) and the ratio of polyunsaturated fatty acids to saturated fatty acids (P/S) peaked at this time.

## 1. Introduction

Baozi is a traditional Chinese delicacy, and mutton is frequently used as a filling. However, raw mutton inherently possesses an unpleasant odor, primarily attributed to its fatty acid composition and the microbial activity in the rumen [1]. Adding carrots to mutton and using it as a filling can enhance the texture of the filling while also elevating its flavor profile. Hot processing techniques can significantly enhance the flavor of meat products, thereby increasing consumer acceptability [2]. Due to the relatively high processing temperatures in conventional stir-frying and roasting, a significant amount of Maillard reaction products (such as heterocyclic compounds and protein melanin) are generated. These compounds can effectively mask and transform the unpleasant smell of mutton odor to a greater extent [3]. However, the temperature during the steaming of baozi is lower compared to that of stir-frying and roasting, making it difficult to mask its odor through basic processing techniques. Previous research has demonstrated that methods of pre-processing, such as rinsing (including changing the rinsing conditions) [4], modifying feeding practices and feed composition (by adjusting the ratio of fatty acids to amino acids) [5,6], and incorporating spices or other exogenous additives (through physical masking or compound interactions) [7], can effectively mitigate undesirable odors. Since the filling is commonly marketed in the form of quick-frozen flour-based products, it must undergo a freezing process to ensure proper preservation and maintain product quality. However, the odor of mutton tends to re-emerge after refrigeration, a challenge that remains difficult to address through pre-processing alone. The primary volatile compounds in spices are terpenes, which typically exhibit low sensory thresholds; thus, even minimal additions can significantly influence odor intensity. In food science, spices and their extracts are frequently employed as antioxidants and flavor enhancers [8]. The incorporation of spices may alter the binding/release capacity of myofibrillar proteins with respect to volatile flavor compounds [9].

Traditional sensory analysis is performed by experienced and professionally trained sensory evaluation panels, which aim to closely align with consumer preferences for the sample. However, due to individual variations in personal preferences, significant discrepancies may arise in the results [10]. Currently, solid-phase micro-extraction gas chromatography–mass spectrometry (SPME-GC-MS) and gas chromatography–ion mobility spectrometry (GC-IMS) are widely used as flavor analysis instruments. Combined with headspace solid-phase microextraction, they can enrich, extract, separate, and detect the odors to be analyzed, as well as separate and detect liquid samples (such as the determination of fatty acid methyl esters) [5,6]. To investigate the changes in odor and fatty acid composition of mutton and carrot filling during reheating, as well as the dynamic evolution of flavor throughout the steaming process, we conducted a comprehensive and systematic analysis and monitoring of the volatile compounds and sensory attributes in mutton and carrot filling.

Based on the results of volatile flavor compounds analyzed by GC-MS and Orthogonal partial least squares discriminant analysis (OPLS-DA) analysis was conducted. Using the data obtained from GC-IMS, fingerprints of volatile compounds in mutton and carrot filling were generated for different steaming times. These findings are anticipated to offer theoretical guidance for identifying the optimal ripening endpoint in samples with distinct odors and elucidating the mechanisms of flavor development.

## 2. Materials and Methods

### 2.1. Sample Preparation

The recipe and process for stuffing in this study were determined by our laboratory’s previous research.

The mutton, carrots, and spices utilized in this study were all procured from Wanda Supermarket in Jinzhou City, Liaoning Province, China. The mutton was rinsed with tap water prior to processing to eliminate blood and debris. The carrots were peeled, and the home food processor (model JR55U-ZM; Hangzhou, China; Supor Co., Ltd.) was utilized to separately smash down the mutton and carrots, which were then set aside for subsequent use. Based on the quality of the mutton, 0.5% thirteen-spices, 3% finely chopped green onion, 1% finely chopped ginger, 5% salt, and 10% Sichuan pepper water (Sichuan pepper:water = 1:7) were incorporated. The aforementioned raw materials were added to the food processor (Hay M5; Qingdao, China; Hanshang Electric Appliance Co., Ltd.) and whipped for 5 min, then set aside. A total of 20 g of the prepared filling was wrapped in 30 g of dough, kneaded, and allowed to proof for 30 min at 37 °C and 75% humidity. After fermentation, the product was subjected to steaming at 100 °C for 8 min, followed by freezing at −18 °C.

### 2.2. Re-Steaming Process

The baozi did not require thawing and could be directly placed in a steamer at 100 °C for steaming of 8, 10, 12, 14, 16, 18, 20, 22, and 24 min. A multi-channel temperature recorder (TP700-8, Shenzhen, China; Top Puri Electronics Co., Ltd.) was used to monitor the temperature throughout the process. After steaming, the baozi is promptly removed and cooled, after which the filling is extracted and ground before being set aside for further use.

### 2.3. Methods

#### 2.3.1. Volatile Compounds Detected by SPME-GC-MS

The SPME-GC-MS method was developed by Yu et al. [11]. A 2.0 g portion of the finely ground sample was transferred to a 20 mL headspace vial, and 3 mL of saturated sodium chloride (analytical grade; Tianjin, China; Bodi Chemical Co., Ltd.) and 10 μL of a 0.62 μg/μL 2-methyl-3-heptanone solution (Aladdin Chemistry, Shanghai, China) were added. The vial was then sealed for analysis. A 75 μm CAR/PDMS (Supelco, Bellefonte, PA, USA) extraction fiber was used for the extraction process, which was balanced at 55 °C for 10 min, followed by a 45 min sorption of VOCs, a 5 min analysis phase at 250 °C, and final detection using the GC-MS (TQ-8040 NX; Kyoto, Japan; Shimadzu Co., Ltd.). Chromatographic column was performed using a Shimadzu DB-5MS capillary column (30 m × 0.25 mm, 0.25 μm film thickness). The temperature program for the column was as follows: an initial temperature of 40 °C held for 3 min, then increased to 70 °C at a rate of 2 °C/min, subsequently raised to 130 °C at 3 °C/min, and finally ramped to 230 °C at 10 °C/min, which was maintained for 5 min. Helium was used as the carrier gas with a constant flow rate of 1 mL/min. Mass spectrometry conditions were set to an ion source temperature of 230 °C, a solvent delay of 2.5 min, and a scanning range of 35–500 *m*/*z*. Volatile organic compounds were identified by matching their mass spectra against the NIST 20 database and calculating the retention index (RI) relative to a homologous series of n-alkanes (C7-C40, purity > 97.0%, procured from St. Louis, MO, USA, Sigma-Aldrich). Semi-quantitative analysis was performed using 2-methyl-3-heptanone (GC pure, ≥98%, procured from Shanghai, China; Aladdin Chemicals) as an internal standard.

#### 2.3.2. HS-GC-IMS Analysis

The GC-IMS analysis method by Liu et al. [12] and the FlavourSpec^®^ IMS instrument (G.A.S., Dortmund, Germany), provided by Shandong Hainen Future Technology Group Co., Ltd., Jinan, China, were utilized for the experimental analysis. A 2 g ground sample was placed into a 20 mL headspace vial. The instrument parameters were set, and the sample was equilibrated at 80 °C for 15 min before being automatically extracted by the sampler system (0.5 mL, 85 °C). Volatile compounds were separated with a capillary column (FS–SE–54–CB–1, 15 m × 0.53 mm × 1.0 μm; purchased from Beijing, China; RESTEK Technology Co., Ltd.), which was used with helium as the carrier gas. The initial flow rate was 2 mL/min with a hold time of 2 min. The flow rate was then increased linearly to 100 mL/min over 8 min, followed by a hold time of 10 min. The total chromatographic run time was 21 min. Using a mixture of n-alkyl ketone (C4–C9, purity > 97.0%, procured from Shanghai, China; Sinopharm Group Chemical Reagent Co., Ltd.) as the standard, standard curves for retention time and retention index were established. The results were compared with the GC retention index database in the VOCal program (NIST. 2020) and the IMS migration time database.

#### 2.3.3. Fatty Acid Analysis

Fat extraction and methylation procedures were refined in accordance with Kang et al.’s [13] methodology. Take 50.00 g of the filling sample, add 100 mL (2:1, *v*/*v*) of a chloroform (analytical reagent, procured from Tianjin, China; Tianli Chemical Reagent Co., Ltd.) -methanol (chromatographic pure, ≥98%, procured from Shanghai, China; Aladdin Chemicals) solution, soak, filter, and then add 20 mL of saturated sodium chloride solution to the filtrate. Mix thoroughly, allow the solution to stand until stratification, and then collect the lower layer. Concentrate the lipid sample by rotary evaporation under a 45 °C water bath.

Fat formulation: Accurately weigh 50 mg of extracted fat and transfer it into a clean, dry test tube. Add 2 mL of a benzene-petroleum (analytical reagent, procured from Tianjin, China; Tianli Chemical Reagent Co., Ltd.) ether mixture (1:1, *v*/*v*) and gently stir to ensure uniform dispersion. Subsequently, add 2 mL of a 14% boron trifluoride methanol solution (analytical reagent, procured from Aladdin Chemicals), mix thoroughly, and incubate in a water bath at 45 °C for 30 min with intermittent shaking to ensure complete reaction. Following incubation, dehydrate the mixture by adding 1 mL of n-hexane (chromatographic pure, ≥98%, procured from Shanghai, China; Aladdin Chemicals) and an adequate amount of saturated sodium chloride (analytical reagent, procured from Tianjin, China; Tianli Chemical Reagent Co., Ltd.). Once the solution has clarified, filter the supernatant through a 0.22 μm organic phase membrane for subsequent analysis.

GC-MS conditions: The inlet temperature was set at 250 °C, with helium as the carrier gas at a flow rate of 1.0 mL/min. The injection volume was 1 μL, and the split ratio was 20:1. The column temperature program was as follows: The initial temperature was held at 140 °C for 2 min, then increased to 200 °C at a rate of 6 °C/min and maintained for 2 min. Subsequently, the temperature was raised to 230 °C at a rate of 2 °C/min and held for 2 min. Finally, it was increased to 250 °C at a rate of 4 °C/min and maintained for an additional 2 min. Mass spectrometry conditions: The interface temperature was 250 °C, the mass-charge ratio scanning range was 25–500 *m*/*z*, the EI ion source temperature was 200 °C, and the solvent delay was 3 min.

### 2.4. Statistical Analysis

All experiments were conducted a minimum of three times, and statistical analysis was performed using SPSS 23.0 (SPSS Inc., Chicago, IL, USA). All data are presented as mean ± standard deviation (SD), with a significance level set at *p* < 0.05. VOCal 0.4.03 software (G.A.S., Dortmund, Germany), which includes GC-IMS Library Search and plugins, was utilized for fingerprint mapping and 2D and 3D odor contour mapping. Cluster heat maps and bar charts were generated using Origin 2025 PRO (Origin Lab Corporation, Northampton, MA, USA). OPLS-DA and VIP analyses were performed and visualized using Sigma14.1 software (Sweden).

## 3. Results

### 3.1. Comprehensive Characterization of VOC Profiles by SPME-GC-MS

**Figure 1:**
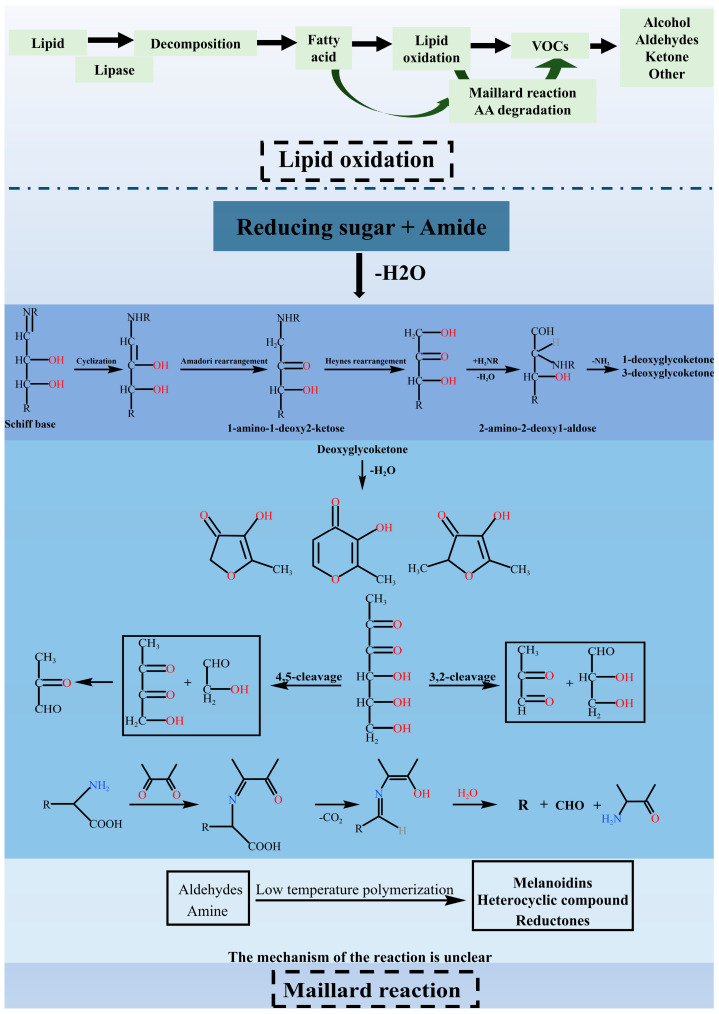
Key flavor pathways of mutton and carrot filling during steaming (lipid oxidation and Maillard reaction).

The carrot and sheep meat filling can develop its distinctive flavor through the thermal degradation of lipids, the Maillard reaction, and their interactions during heating. Figure 1 provides a clearer illustration of this process.

**Figure 2:**
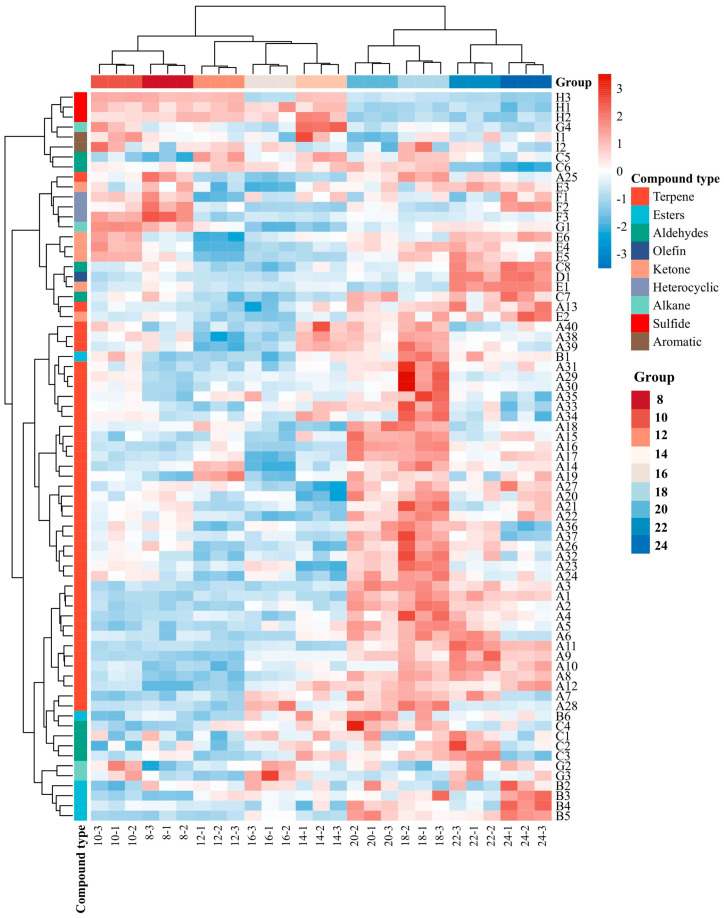
Heat map clustering analysis of volatile compounds in mutton and carrot fillings with different steaming times by the SPME-GC-MS method.

A total of 73 volatile compounds were identified in fillings with different steaming times using SPME-GC-MS technology, as shown in Figure 2. The detailed information of 73 compounds, including their numbers, compound names, CAS numbers, and retention indices (RI), is presented in Table 1. Among these, terpenes were the most abundant, comprising 40 species, followed by aldehydes (8), esters (6), ketones (6), alkanes (4), sulfides (3), heterocyclic compounds (2), aromatic compounds (2), and alkene (1). These compounds were detected across all steaming time groups. No volatile substances of taint such as 3-methylindole and 4-methylphenol were identified in the sample, indicating that the taint of the sample in this study was small, which may be due to the addition of spices and carrots, which contain more aromatic compounds and can cover the smell, or the spatial structure of the compounds can adsorb the smell of mutton. To a certain extent, the compounds were embedded. 2-sec-Butyl-3-methoxypyrazine, a characteristic compound of raw carrots, is undetectable at all cooking times, suggesting that the carrots have undergone complete ripening [14].

Among the 40 terpene compounds identified, sabinene exhibited the highest content across all groups, with a concentration of 5644.88 ± 826.17 μg/kg when the steaming time was 20 min. The content of sabinene increased from 8 to 20 min and decreased after 20 min. Secondly, β-caryophyllene (A13) had the second-highest content after sabinene (A35), and both compounds belong to the characteristic flavor compounds in ginger. Carrots contained higher levels of monoterpenes and sesquiterpenes, such as α/β pinene (A9/A23) and myrcene (A12) [15]. It is inferred that the majority of terpenes were introduced through the addition of spices, while only a small number were present in the raw materials.

Aldehydes are significant volatile compounds in ruminants, serving as intermediates in the Maillard reaction and the thermal degradation of lipids, and also play a role in the interaction of the amino-carbonyl reaction [16]. These compounds are primarily derived from the thermal degradation and secondary reactions of fats. These compounds typically exhibit a low odor threshold, enabling them to significantly contribute to the aroma even at relatively low concentrations [17]. Most aldehydes impart a meaty flavor; however, high concentrations can lead to an unpleasant rancid fatty acid smell. Octanal (C2), heptanal (C5), and 1-nonanal (C1) are formed during the oxidation of lipids, particularly through the breakdown of oleic acid and other related processes [18]. As shown in Figure 2, the content of octanal (C2) reached its peak at a cooking time of 24 min. In contrast, the lowest levels were observed at 10, 12, and 16 min, with no significant differences among these three time points (*p* > 0.05). After 16 min, the concentration of octanal (C2) exhibited an increasing trend, which may be attributed to the effect of continuous high temperature [19].

Heptanal (C5) reached its peak concentration at both 22 and 24 min, with no statistically significant difference between these two time points. Between 8 and 20 min, the concentration remained relatively stable but was significantly lower, approximately halving when compared to the levels observed at 22 min and thereafter. The change in 1-nonanal (C1) (grassy flavor) during the cooking process is similar to that of octanal (C2) and heptanal (C5), with peak concentration observed at 24 min and the lowest content recorded at the endpoint of steaming. Prior to 16 min of steaming, the overall content shows a relatively decreasing trend, while after the endpoint, it exhibits an increasing trend. These changes can be attributed to alterations in the binding capacity between myofibrillar proteins and flavor compounds, as well as the oxidation of fatty acids. During heating, the binding capacity of myofibrillar proteins with flavor compounds increases as temperature rises [20]. However, after prolonged heating, the degradation and spread of the MPs’ structure result in a diminished binding capacity of MPs to the compounds, leading to further volatilization of some compounds [21]. Heptanal (C5) is a product of fatty acid oxidation, and heating undoubtedly accelerates this process [22,23]. Nevertheless, different compounds exhibit varying oxidation rates. As a significant aldehyde produced during lipid oxidation in meat, the progressive increase in hexanal content is likely attributed to the gradual oxidation of unsaturated fatty acids (UFAs) during heating [24].

Ketones are also the products of fat oxidation, primarily resulting from the oxidation of fatty acids or the catabolism of amino acids. Due to their distinctive flavor, ketones are believed to influence the flavor profile of meat products [25]. The 2-heptanone (E5) exhibits a pear-like aroma. Resconi et al. [26] demonstrated that 2-heptanone (E5) is a crucial component of the volatile flavor profile in mutton products and can serve as a key indicator for assessing product deterioration. As illustrated in Figure 2, the content of acetophenone, which has a bitter almond taste, decreased significantly between 8 and 12 min (*p* < 0.05), remained unchanged between 12 and 18 min (*p* > 0.05), and showed a slight increasing trend after 20 min, similar to that of 1-nonanal (C1). The content of 2-nonanone (E1) was the highest among all ketones, reaching a peak value of 69.04 ± 4.00 μg/kg at 12 min, whereas its content was significantly lower at both 8–16 min. The concentration of Maillard reaction products does not consistently increase with heating time; instead, it alters the final equilibrium of flavor compounds, thereby influencing the flavor characteristics. Therefore, the changes in the content of certain compounds do not follow a uniform trend.

Only six esters were detected by GC-MS. Under normal conditions, ester compounds are formed through the oxidation of lipids and the interaction of alcohols derived from free fatty acids. These compounds typically exhibit a sweet or fruity flavor, with the fruity aroma primarily resulting from the esterification reaction between alcohols and acids [27]. In this study, three long-chain esters and three terpene esters were identified: ethyl hexanoate (B2), ethyl caprylate (B3), dibutyl phthalate (B1), bornyl acetate (B4), terpinyl acetate (B5), and linalyl acetate (B6). These compounds, primarily derived from ginger and other flavorings, were all detected between 8 min and 24 min. Ethyl caprylate (B3) has a fruity and brandy aroma, while ethyl hexanoate (B2) has a fruity aroma. At the 14th min of steaming, the highest content of ethyl caprylate (B3) was 40.96 μg/kg, and the highest content of ethyl caproate (B3) was 265.14 μg/kg, which may be because in linear ester compounds, the longer the carbon chain, the greater the degree of binding to proteins [28].

Among the heterocyclic compounds identified, two furans and one pyrazine compound were detected: 2-(3-methyl-2-butenyl)-3-methylfuran (F1), 3-(4-methyl-3-amyl) furan (F2), and 3-Ethyl-2,5-dimethylpyrazine (F3). These compounds are essential flavor components in meat products, primarily formed through the Maillard reaction, heterocyclic reactions, and the thermal degradation of lipids during the final stage of Strecker degradation, as well as the interactions among these processes [29,30]. The 2-(3-methyl-2-butenyl)-3-methylfuran (F1), commonly referred to as Rose furan, is a furan heterocyclic compound with a fresh lemon aroma. The concentration of Rose furan reached its peak at 24 min of steaming, while within the range of 8–22 min, the concentration exhibited a slight but statistically insignificant increase (*p* > 0.05). 3-(4-methyl-3-amyl) furan (F2) is a furan monoterpene compound characterized by its floral and orange fragrance. The concentration of this compound was observed to increase significantly following a 20–24 min heating period; however, no significant changes were noted steaming for 8–18 min (*p* > 0.05). 3-Ethyl-2,5-dimethylpyrazine (F3), which is utilized as a flavor and fragrance in food due to its cocoa and nut-like aroma, exhibited no significant change in content during the 8–20 min cooking period but showed a slight upward trend. After 20 min, the content of 3-Ethyl-2,5-dimethylpyrazine (F3) decreased significantly (*p* < 0.05).

In this study, only three types of sulfides were identified using GC-MS; however, their concentrations were relatively high. They originate from the continuous heating and thermal activation processes of scallions or mutton. The concentration of propyl disulfide (H1) peaked at the 14th min, with a significant or non-significant decrease observed both before and after this time point, though it is not increasing. This phenomenon can be attributed to the intrinsic high volatility of sulfides. Heating triggers the release of sulfide compounds; however, prolonged exposure to elevated temperatures can result in sulfides either adhering to the crust or volatilizing into the air, thereby reducing their concentration within the filling. The concentrations of methyl propyl sulfide (H2) and (E)-1-propenyl propyl disulfide (H3) are comparable to that of propyl disulfide (H1), yet the inflection points for their content changes differ.

The concentrations of the four detected alkanes are relatively low, and they are all long-chain alkanes. Most long-chain alkanes are derived from the degradation of ester compounds. Due to their high threshold, these alkanes generally exhibit weak aromas or are odorless. While they have a certain modifying effect on meat products, their contribution to the overall flavor is minimal [31].

### 3.2. OPLS-DA Analysis

**Figure 3:**
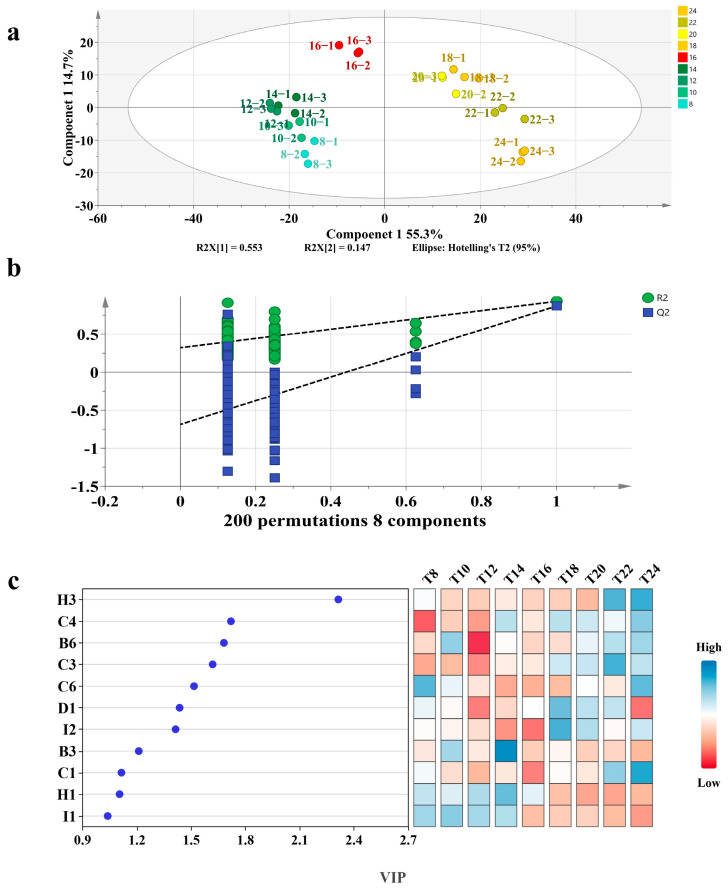
OPLS-DA model of volatile compounds in mutton and carrot stuffing samples. (**a**) Orthogonal partial least squares discriminant analysis score map of different samples (8–24, indicating the steaming time of 8–24 min, respectively); (**b**) confidence test results of different samples; (**c**) VIP values of different samples (T8T24, indicating the steaming time of 824 min, respectively).

To further investigate the flavor differences in mutton and carrot fillings under varying steaming times, SPME-GC-MS data were analyzed using the OPLS-DA statistical method. To eliminate the potential influence of spices (terpenes) on the identification outcomes, terpenes were excluded from the OPLS-DA analysis. Therefore, using 33 shared aroma components as the dependent variable and steaming time as the independent variable, OPLS-DA analysis (Figure 3a) effectively distinguishes lamb and carrot stuffing samples based on different steaming times. In this analysis, the independent variable fitting index (R^2^x) is 0.967, the dependent variable fitting index (R^2^y) is 0.921, and the model prediction index (Q2) is 0.82. Since both R2 and Q2 exceed 0.5, these results indicate that the model fitting is reliable. After 200 permutation tests, as illustrated in Figure 3b, the y-intercept of the Q2 regression line is negative, indicating that the model does not exhibit overfitting and the validation is effective. Therefore, the results can be utilized for the identification and analysis of mutton and carrot fillings across various steaming durations. The VIP value of VOCs serves as an indicator to assess the impact and explanatory power of each variable factor on the classification and discrimination of sample groups. A higher VIP value indicates a greater inter-group difference in VOCs, thereby enhancing its significance in sample discrimination and classification. The VIP values for various volatile flavor compounds are presented in Figure 3c. According to the criteria of *p* < 0.05 and VIP > 1, a total of 11 distinct aroma compounds in mutton and carrot fillings were identified, comprising esters (2), aldehydes (4), olefin (1), sulfide (2), and aromatic compounds (2).

### 3.3. GC-IMS Analysis

**Figure 4:**
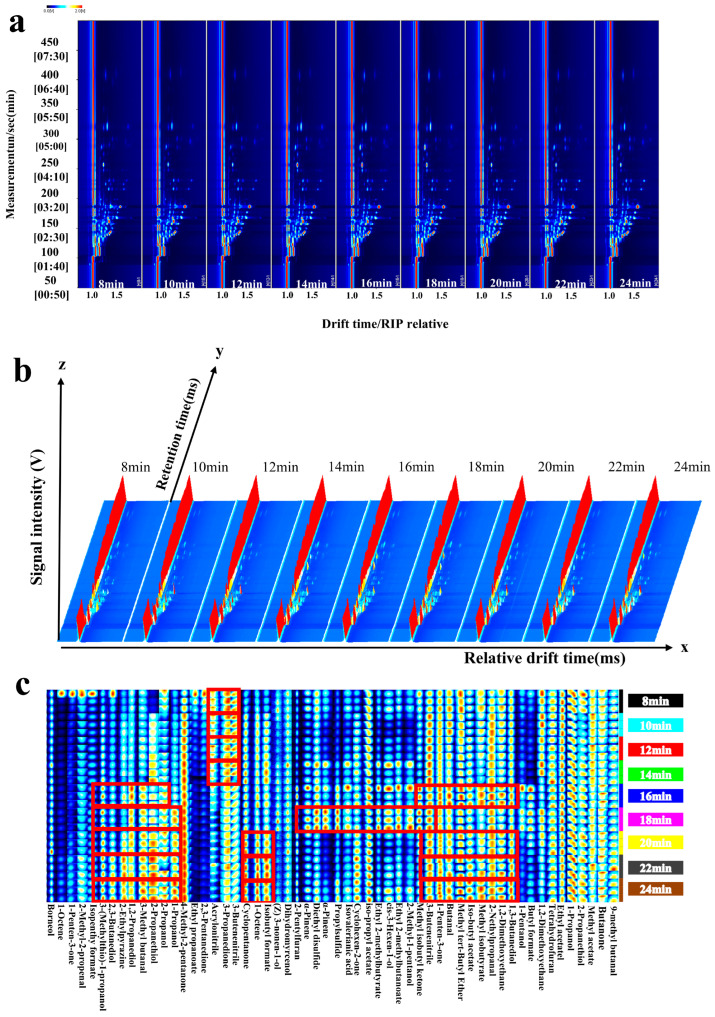
Analysis of volatile compounds in mutton and carrot fillings. (**a**) The 2D topographic maps; (**b**) 3D topographic maps; and (**c**) fingerprints.

As shown in Figure 4a, a total of 44 compounds (excluding dimers and trimers) were identified by GC-IMS, including aldehydes (3), ethers (1), esters (10), sulfur-containing compounds (2), ketones (7), alcohols (10), olefins (3), heterocyclic compounds (3), acids (1), and other compounds (4). Among these, esters and alcohols are the predominant compounds detected by GC-IMS, whereas GC-MS primarily detects terpenes, likely due to their distinct detection principles [32].

To better understand the variations in VOC concentrations within mutton and carrot filling samples subjected to different steaming durations, 2D and 3D topographic maps of VOC distributions were constructed (Figure 4a,b). The yellow and red regions represent the differences in VOC concentrations across various samples, with darker shades of red indicating higher concentration levels. As shown in the GC-IMS 2D plot (Figure 4a), the retention time of flavor compounds across all sample groups ranged from 100 to 450 s, with ion drift times between 1.0 and 2.0 milliseconds. The 3D graph reveals that as the steaming process progresses, both the number and intensity of the VOCs change, indicating that steaming modifies the types and concentrations of VOCs in the mutton and carrot filling. This is likely attributed to heating-induced lipid oxidation and the Maillard reaction, which alter the interactions among these compounds.

To more intuitively analyze the impact of steaming time on the changes in VOCs of mutton and carrot filling, characteristic peaks under different steaming times were selected, and a fingerprint was generated using the Gallery Plotbuilt in the GC-IMS system (Figure 4c). The red rectangles indicate the characteristic flavors at different steaming times.

The contents of 12 compounds, such as 1-octene and 1-pentene-3-ketone, increased with the progress of steaming. The contents of these compounds, which were mainly alcohols, increased with the extension of heating time. Alcohol compounds are another product of lipid oxidation; they have a low threshold and mainly originate from the decomposition of secondary hydroperoxides of fatty acids. However, because they generally have a high threshold, their contribution to the flavor of mutton is considered not too important [25]. The contents of five compounds, such as ethyl propionate and 2,3-pentanedione, decreased with the progress of steaming, which could be explained by the physicochemical properties of the compounds.

In the analysis of fingerprint data, the entire steaming process (8–24 min) can be divided into three distinct phases: the early stage of steaming (8–14 min), the steaming endpoint (16 min), and the late stage of steaming (18–24 min). As shown in the fingerprint (Figure 4c), the primary VOCs during the early stage of steaming were tetrahydrofuran and 2-propyl mercaptan, which are responsible for the onion flavor. The concentrations of these two compounds decreased as the process time extended, and a significant reduction in red brightness was observed starting at 16 min. The characteristic VOCs at reach the end of steaming (16 min) include methyl isobutyl ketone, 1-allyl-3-ketone, 2-butanone, tert-butyl methyl ether, isoamyl formate, methyl isobutyrate, 2-methylpropanal, 1,2-dimethoxyethane, 1,3-butanediol, isopropanol, 3-methylbutanal, 1,3-butanal, 2-propanediol, 2-ethylpyrazine, 2,3-butanediol, and 3-methylsulfanylpropan-1-ol. This indicates that the flavor of the filling sample changes slowly and to a lower degree after being steamed for 16 min, and the main reason for this difference is the process of lipid oxidation. Most of the above substances are products of the Maillard reaction or lipid oxidation and secondary metabolites, and their content is increased after the steamed period, which is in line with the conditions for the occurrence of the Maillard reaction. For instance, 2-ethylpyrazine is generated through the further reaction and cyclization of carbonyl compound intermediates that arise from the Strecker degradation of amino acids during the Maillard reaction. Additionally, 3-methylbutyraldehyde undergoes thermal decomposition into free fatty acids, and subsequent oxidation of unsaturated fatty acids leads to the formation of peroxides, which are then further decomposed. The fingerprint reveals a distinct change in VOCs during the 16–18 min steaming period, which aligns with the SPME-GC-MS results.

As previously discussed, owing to the distinct principles underlying GC-MS and GC-IMS, the VOCs identified by these two techniques exhibit nearly complete dissimilarity. Specifically, GC-MS predominantly detects terpene compounds and aldehydes, with limited detection of esters and ketones. Conversely, GC-IMS primarily identifies esters and alcohols, with lesser capability for detecting terpenes. This demonstrates that by leveraging the principal characteristics of both instruments, we can collaboratively analyze the VOCs in the sample, thereby enhancing and complementing the data [33].

A total of 116 VOCs were identified using GC-MS and GC-IMS, including terpenes (41), alcohols (10), aldehydes (10), ketones (13), esters (16), sulfides (5), heterocyclic compounds (6), aromatic compounds (2), and others (13). In addition to terpenes, which are predominantly associated with spices, the predominant VOCs in this study’s sample are ketones and esters, both of which have relatively high detection thresholds.

### 3.4. Fatty Acids Analysis

During processing and cooking, lipids undergo thermal degradation through a series of reactions, including dehydration, decarboxylation, hydrolysis, dehydrogenation, and C-C bond cleavage under anaerobic conditions. These reactions result in the formation of free fatty acids, saturated hydrocarbons, unsaturated hydrocarbons, β-ketoacids, and esters. Notably, the degradation of unsaturated fatty acids represents a crucial pathway for generating volatile compounds in meat products.

Specifically, oleic acid undergoes oxidation and degradation to produce heptyl aldehyde, octyl aldehyde, and nonanal, which impart fruity and fatty flavors. Likewise, the oxidative degradation of linoleic acid leads to the formation of valeraldehyde and hexanal. Moreover, the oxidative degradation of linolenic acid results in the production of benzaldehyde and amyl alcohol.

**Figure 5:**
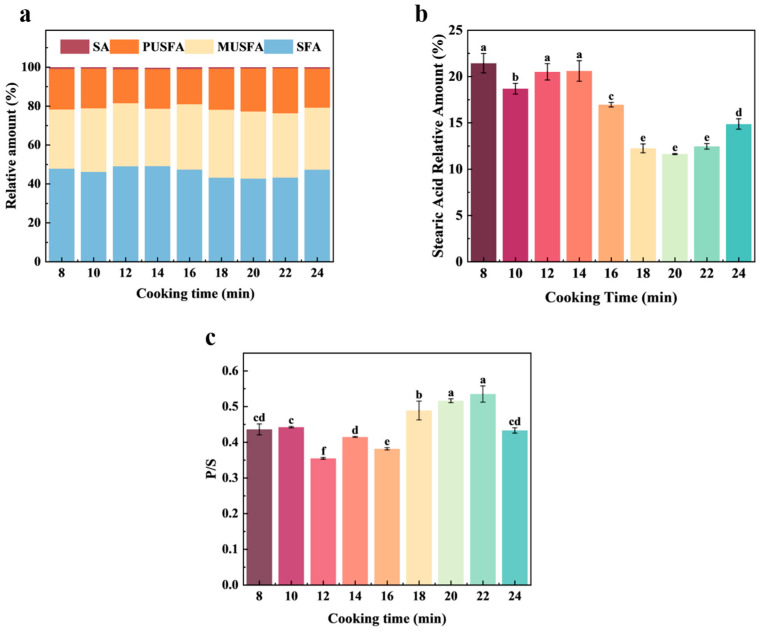
Analysis of free fatty acids in mutton and carrot fillings. (**a**) Histogram of the content of different types of fatty acids; (**b**) histogram of stearic acid content; (**c**) bar chart of P/S values. (**a**–**e**) significant differences were observed among different time points (*p* < 0.05).

During the steaming process, a total of 33 types of free fatty acids were identified in the mutton and carrot fillings, including saturated fatty acids (SFAs, 7), monounsaturated fatty acids (MUFAs, 8), polyunsaturated fatty acids (13), and substituent fatty acids (5) (Figure 5a). The contents of oleic acid (C18:1), stearic acid (C18:0), palmitic acid (C16:0), and linoleic acid (C18:2) were relatively high among the 33 kinds of free fatty acids detected. This result is consistent with the findings of Ding et al. [34] regarding fatty acids in mutton. SFAs can serve as an energy source for the human body; however, excessive consumption of SFAs can lead to obesity and contribute to various complications. Dietary intake of SFAs has been associated with an increased risk of coronary heart disease and other health issues. Various fatty acids can mediate endothelial dysfunction through distinct mechanisms, thereby playing a role in the development of atherosclerosis. Liu et al. [35] investigated the association between the thickness of the coronary plaque fibrous cap and levels of palmitic acid and arachidonic acid. Their findings revealed a statistically significant difference between the groups (*p* < 0.001), indicating that the thickness of the coronary plaque fibrous cap might be associated with saturated fatty acids.

The 4-alkyl branched-chain fatty acids, the main substances that form mutton taint, were not detected in the fillings during all cooking times in this study, indicating that the addition of spices and carrots was able to remove most of the taint. In addition to the smell of mutton from 4-alkyl branched-chain fatty acids, stearic acid is also one of the important components in mutton. It circulates through the blood and accumulates in the fat [3,36]. The relative content of stearic acid generally decreased during the 8–18 min period, and there was no significant difference at 18–22 min, but there was a slight increase at 24 min (*p* < 0.05) (Figure 5b). This shows that the heating process can reduce the accumulation of mutton taint substances, but as the heating time continues to increase, the oxidation of large molecules, such as fats and proteins, and their undesirable odor may reappear.

Among all fatty acids, SFAs and MUFAs are the most abundant, with stearic acid and palmitic acid being the predominant types. This is primarily attributed to the higher levels of stearic acid in ruminants compared to other animals, which significantly contributes to their distinct odor [13]. Among saturated fatty acids, arachidonic acid was detected only within the steaming time range of 8–10 min, with its relative content showing a decreasing trend. The relative content of stearic acid remained relatively stable between 8 and 14 min but gradually decreased from 16 to 24 min. This phenomenon may be attributed to the fact that, although the filling is fully cooked after 16 min, stearic acid continues to decompose due to prolonged heat exposure or undergoes esterification reactions with alcohol compounds, leading to a reduction in its concentration.

The content of oleic acid is the highest among UFAs, followed by linoleic acid. Studies have shown that the content of oleic acid is positively correlated with the intensities of odor and flavor [36]. In this study, the content of oleic acid showed a trend of first decreasing and then increasing, and there was no significant difference at the 20th min (*p* > 0.05).

The type and proportion of fatty acids affect the nutritional value and flavor of meat, which is of great significance to human health. Fatty acids were mainly evaluated by the ratio of P/S and the contents of n-6 and n-3 series PUFAs. Higher P/S ratios result in more tender and juicy meat, as well as higher flavor scores. [37]. In this study, the P/S content reached its peak when the cooking time was between 20 and 22 min, and the levels of both n-6 and n-3 series PUFAs were also at their highest (Figure 5c).

## 4. Discussion

Multivariate statistical analysis demonstrates that GC-MS and GC-IMS technologies can reliably detect and identify VOCs in mutton and carrot fillings at various steaming durations. In comparison with other studies utilizing GC-MS and GC-IMS, terpene compounds were predominantly detected by GC-MS in this study, whereas esters, aldehydes, and alcohols were primarily identified by GC-IMS. Ketone compounds were detected to varying extents by both techniques. The variations in these VOCs are the primary factors contributing to the distinct odor profiles of mutton and carrot fillings during the steaming process. A total of 33 types of fatty acids were identified during the steaming process. It is noteworthy that 4-alkyl branched-chain fatty acids, which are considered a critical factor in the development of off-flavors, were not detected at any stage of the steaming process. Conversely, stearic acid exhibited a dynamic pattern characterized by an initial decrease followed by an increase during the steaming process. These findings suggest that the system under investigation inherently possesses the capacity to mitigate the formation of off-flavors. Furthermore, the dynamic changes in fatty acids throughout the steaming process can serve as a crucial reference indicator for optimizing the thermal processing parameters of mutton. By employing the P/S value as an evaluation index for aroma quality, we observed that the P/S value reached its maximum when the steaming time was between 20 and 22 min. However, in the current research, there are not many studies that use this evaluation method. The results of the comprehensive odor analysis indicate that, in comparison to steaming for 16 min, extending the steaming duration to 20–22 min not only ensures the stability of stearic acid content but also effectively preserves the flavor of meat and spices. Within the food industry, under relatively consistent conditions (e.g., maintaining a controlled ratio of dough to filling), a steaming time of 20–22 min can serve as a standardized parameter for this process. Nevertheless, this study is subject to certain limitations, such as constraints imposed by detection technology. For instance, it is challenging to achieve comprehensive and precise separation and detection of diverse compound types using a single device. Consequently, we propose that future research could integrate well-trained sensory panels for subjective evaluation alongside advanced analytical techniques to further explore the correlation between VOCs and olfactory/gustatory perception. This approach would enable dynamic assessment of food processing from the perspective of consumer subjective acceptance.

## 5. Conclusions

In this study, GC-MS and GC-IMS technologies were employed to systematically investigate the variation patterns of volatile components in mutton and carrot fillings under different cooking durations. The findings indicate that differences in VOCs, such as terpenoids, esters, aldehydes, alcohols, and ketones, are the critical factors contributing to the dynamic changes in flavor characteristics during the steaming process. GC-MS demonstrated relatively high detection sensitivity for terpenoids, whereas GC-IMS exhibited superior resolution in detecting esters, aldehydes, and alcohols. The complementary nature of these two techniques effectively elucidated the dynamic change of the laws of volatile components. Regarding fatty acid composition, a total of 33 types of fatty acids were identified, with no detection of 4-alkyl branched-chain fatty acids. Stearic acid exhibited a trend of initially decreasing and subsequently increasing during the cooking process, which may be closely associated with the dynamic equilibrium between fat hydrolysis and oxidation reactions. By evaluating the P/S ratio, it was determined that within the steaming period of 20–22 min, the P/S value reached its peak, at which point the flavor quality of the sample was optimal. Controlling the steaming and boiling time within the range of 20–22 min not only maintains the stability of stearic acid content but also better preserves the characteristic flavors of mutton and spices. In conclusion, optimizing the steaming and boiling time to 20–22 min significantly enhances the flavor quality of mutton and carrot fillings, providing a theoretical foundation for the advancement of food processing techniques. Future research could delve deeper into the relationship between volatile components and sensory properties, thereby facilitating the effective utilization of flavor characteristics in special-flavored foods.

## Figures and Tables

**Table 1:** SPME-GC-MS analysis results—code, compound name, CAS number, and retention index (RI).

Code	Volatile Compounds	CAS	RI Literature	RI Calculation
A1	terpinolene	586-62-9	1090	1081
A2	(+)-7-epi-sesquithujene	159407-35-9	1389	1380
A3	2-isopropyl-5-methylanisole	1076-56-8	1235	1245
A4	4-allylanisole	140-67-0	1204	1213
A5	(+)-citronellal	2385-77-5	1154	1154
A6	(E)-citral	141-27-5	1272	1269
A7	β-citral	106-26-3	1241	1243
A8	anethole	4180-23-8	1280	1284
A9	α-pinene	80-56-8	937	934
A10	α-phellandrene	99-83-2	1002	1011
A11	camphene	79-92-5	943	951
A12	myrcene	123-35-3	990	998
A13	β-caryophyllene	87-44-5	1410	1415
A14	α-curcumene	644-30-4	1482	1486
A15	β-bisabolene	495-61-4	1506	1514
A16	(-)-α-copaene	3856-25-5	1375	1372
A17	(E)-γ-bisabolene	53585-13-0	1534	1538
A18	(E)-α-bisabolene	25532-79-0	1508	1504
A19	ocimene	13877-91-3	1053	1050
A20	β-sesquiphellandrene	20307-83-9	1526	1523
A21	α-caryophyllene	6753-98-6	1460	1460
A22	(E)-β-farnesene	18794-84-8	1459	1459
A23	β-pinene	18172-67-3	973	978
A24	D-camphor	464-49-3	1154	1154
A25	α-bergamotene	17699-05-7	1442	1430
A26	(+)-cyclosativene	22469-52-9	1370	1371
A27	dihydrocurcumene	1461-02-5	250	250
A28	(E)-germacrene D	23986-74-5	1480	1480
A29	carvone	6485-40-1	1246	1249
A30	fenchone	1195-79-5	1091	1086
A31	(+)-Δ-cadinene	483-76-1	1528	1519
A32	α-ylangene	14912-44-8	1372	1372
A33	γ-terpinene	99-85-4	1065	1061
A34	(±)-β-copaene	18252-44-3	1433	1433
A35	sabinene	3387-41-5	973	980
A36	(E, E)-3,6-α-farnesene	502-61-4	1508	1514
A37	5-(1,5-Dimethyl-4-hexenyl)-2-methyl-1,3-cyclohexadiene	495-60-3	1493	1493
A38	γ-curcumene	451-55-8	1485	1479
A39	β-(Z)-ocimene	3338-55-4	1106	1109
A40	1-methyl-4-isopropyl-1-cyclohexen-3-one	89-81-6	1259	1250
B1	phthalic acid dibutyl ester	84-74-2	1963	1960
B2	ethyl hexanoate	123-66-0	996	996
B3	ethyl caprylate	106-32-1	1194	1191
B4	L-bornyl acetate	20347-65-3	1300	1304
B5	terpinyl acetate	80-26-2	1352	1358
B6	linalyl acetate	115-95-7	1253	1251
C1	1-nonanal	124-19-6	1104	1109
C2	octanal	124-13-0	1123	1106
C3	benzaldehyde	100-52-7	967	969
C4	1-decylaldehyde	112-31-2	1205	1201
C5	heptanal	111-71-7	903	909
C6	hexanal	66-25-1	805	803
C7	3,7-dimethyl-3,6-octadienal	55722-59-3	1191	1185
C8	phenylacetaldehyde	122-78-1	1041	1046
D1	(Z)-2-thia-3-pentene	52195-40-1	731	731
E1	2-nonanone	821-55-6	1090	1095
E2	3-heptanone	106-35-4	894	894
E3	5-nonanone	502-56-7	1059	1051
E4	3-methyl-2-heptaone	2371-19-9	930	931
E5	2-heptanone	110-43-0	892	892
E6	acetophenone	98-86-2	1330	1312
F1	2-(3-methyl-2-butenyl)-3-methylfuran	15186-51-3	1116	1104
F2	3-(4-methyl-3-amyl) furan	539-52-6	1118	1102
F3	3-ethyl-2,5-dimethylpyrazine	13360-65-1	1079	1084
G1	tetradecane	629-59-4	236	236
G2	n-hendecane	1120-21-4	1027	1022
G3	decane	124-18-5	1039	1031
G4	2-methyldecane	6975-98-0	1088	1076
H1	propyl disulfide	629-19-6	1103	1105
H2	1-(methylsulfanyl)propane	3877-15-4	752	752
H3	(E)-1-propenyl propyl disulfid	23838-21-3	1117	1119
I1	benzene	71-43-2	662	662
I2	4-isopropyltoluene	99-87-6	1033	1027

## Data Availability

The original contributions presented in the study are included in the article, further inquiries can be directed to the corresponding author.
